# Synergic effect of diet and polysaccharide-based complex administration in the treatment of children and adolescents with metabolically unhealthy obesity: the Polymets Study

**DOI:** 10.1007/s40618-026-02860-0

**Published:** 2026-03-14

**Authors:** Giulia Fiore, Asli Altayeva, Simona Panelli, Diego De Zan, Lodovico Sterzi, Valeria Calcaterra, Martina Tosi, Franco Folli, Gianvincenzo Zuccotti, Francesco Comandatore, Elvira Verduci

**Affiliations:** 1 Department of Pediatrics, Vittore Buzzi Children’s Hospital, University of Milan, Milan , Italy; 2 Department of Health Sciences, University of Milan, Milan, Italy; 3 Department of Biomedical and Clinical Sciences, Pediatric Clinical Research Center Romeo and Enrica Invernizzi, University of Milan, 20157 Milan, Italy; 4 Department of Internal Medicine, University of Pavia, 27100 Pavia, Italy; 5 Department of Biomedical and Clinical Sciences, University of Milan, Milan, Italy; 6 Pediatric Unit, Foundation IRCCS Ca’ Granda Ospedale Maggiore Policlinico, Milan, Italy

**Keywords:** Children, Childhood obesity, Gut microbiota, Butyrate, Fat mass

## Abstract

**Purpose:**

The primary aim of the Polymets Study is to evaluate the effect on gut microbiota composition of a polysaccharide-based complex administration combined with dietary and lifestyle interventions in a group of children and adolescents with metabolically unhealthy obesity (MUO).

**Methods:**

In this clinical trial, children and adolescents (8–14 years) with obesity (defined as body mass index >  + 2 standard deviation score [BMI SDS] according to World Health Organization) and cardio-metabolic alteration (hypertriglyceridemia, hypertension, hypo-HDL cholesterol, altered glucose metabolism) were enrolled. Participants received from baseline (T0) to 4 months (T1) combined polysaccharide-based complex administration (5 g/day mixture of soluble and insoluble fibres) and Mediterranean diet intervention; from 4 to 8 months (T2) they underwent dietary intervention alone. At T0, T1 and T2 gut microbiota analysis, body composition assessment, blood tests and Mediterranean diet adherence (KIDMED score) were assessed.

**Results:**

Overall, 31 children were enrolled (10.9 ± 1.6 years, F/M 8/23). BMI SDS significantly decreased at each timepoint (p < 0.001), whilst fat mass% significantly decreased only at T1 (p = 0.035), vs T0. The Principal Component Analysis showed a trend of reduced dispersion at the phylum taxonomic level at T1, having *Firmicutes*, *Bacteroidota* and *Proteobacteria* as major contributors to the variance. At T1, there was an enrichment in *Barnesiellaceae* family, *Erysipelotrichaceae_UCG-003 *and *Parabacteroides* genera (p < 0.05), vs. T0. At T2 *Oscillospiraceae_unclassified* and *Clostridia_unclassified* increased significantly, whereas *Erysipelotrichaceae_UCG − 003* and *Escherichia_Shigella* decreased significantly (p < 0.05), vs T1.

**Conclusion:**

Polysaccharide-based complex intervention synergistically contributes for enrichment in microbial marker of metabolic health and body adiposity reduction, while the subsequent solo-diet phase triggers expansion of butyrate-producing lineages and overall reduction of pathogenic taxa.

**Supplementary Information:**

The online version contains supplementary material available at 10.1007/s40618-026-02860-0.

## Introduction

Obesity is a global public health problem and is recognized as a systemic, chronic, low-grade inflammatory disease that contributes to increased metabolic and cardiovascular risks both in childhood and adulthood [1–3]. Globally, over one billion people are living with obesity. In the WHO European Region, the latest European Childhood Obesity Surveillance Initiative (COSI) data indicate that 25% of children aged 7–9 years have overweight, including 11% with obesity [4, 5]. Multi-component behavioural interventions (targeting diet, physical activity, and psychological health) are first line treatments for childhood obesity [6–8], but often fail to achieve significant and clinically meaningful reduction of overweight and obesity. Specifically, compliance with these multifactorial interventions is very difficult to be maintained in the long term [9–11].

Human gut microbiota is a complex community of microorganisms [12, 13], and Turnbaugh et al. first demonstrated the causal link between gut microbiota composition and obesity in preclinical model [14]. Consistently, obesity is associated with lower gut bacterial diversity compared to normal-weight status both in adults and children [12, 15, 16]. In addition, bacterial fermentation product, mainly short-chain fatty acids (SCFAs), could affect satiety and metabolic health [17, 18]. Accordingly, gut microbiota-based interventions are being investigated as potential targets for childhood obesity treatment. A recent meta-analysis by Fahim et al. found that prebiotic supplementation [e.g., inulin, galactooligosaccharides (GOS), fructo-oligosaccharides (FOS)] produces modest reductions in body mass index (BMI) and body weight in children and adolescents [19], whereas evidence on other dietary fibres and non-digestible carbohydrates on gut microbiota in paediatric age groups remains limited.

Dietary fibres and non-digestible carbohydrates include disaccharides, oligosaccharides (e.g. FOS, GOS) and polysaccharides (inulin, cellulose, hemicellulose, pectin, mucilages, resistant starch) which perform different functions in the gut [20]. Several mechanisms are described by which dietary fibres modulate satiety in pre- and post-absorbing phase, and decelerate the gastric emptying rate and the stimulus to the production of hormones involved in appetite regulation [21, 22]. In addition, improving satiety, fibre ingestion could determine a lower caloric intake at the next meal, and, in the long term, it could be effective also in weight management [23].

Administration of fibre-rich supplements with gel-forming properties, composed of polysaccharide-based macromolecules, acts through the generation of a network of soluble and insoluble fibres that are typically not absorbed by the body. This mechanism aims to reduce the absorption of carbohydrates and lipids from the diet, thereby decreasing the extent and rate of postprandial glycaemic increase and levelling the concentrations of glucose and insulin in the blood [23–25]. Previously, one randomized, double-blind, placebo-controlled trial showed a significantly lower postprandial increase in triglycerides, in children with obesity after an intervention with polysaccharide-based complex [26]. The same intervention combined with a low-glycemic index dietary treatment demonstrated greater reductions in BMI z-scores and insulin resistance compared to diet alone [27]. To date, no studies are available that demonstrate whether polysaccharide-based complex can impact the gut microbiota of children and adolescents with obesity.

Based on these considerations, the primary aim of this clinical trial is to evaluate the effect on gut microbiota composition of a polysaccharide-based complex administration combined with dietary and lifestyle interventions in a group of children and adolescents with metabolically unhealthy obesity (MUO). Furthermore, the study aims to evaluate the effectiveness of this intervention in improving obesity-related parameters and the overall cardiometabolic profile.

## Materials and methods

### Settings and participants

In this clinical trial, children and adolescents with MUO were enrolled at the Department of Pediatrics of the Vittore Buzzi Children’s Hospital, Milan, Italy from February 2022 to November 2023. The Polymets Study was conducted according to the guidelines of the Declaration of Helsinki and was approved by the Ethical Committee (CET Milano Area 1 n. 2021/ST/260) and registered in ClinicalTrails.gov. After being informed about the study, written consent was collected from caregivers and participants. Inclusion criteria were defined as follows: age 8 to 14 years; obesity defined as BMI z-score ≥  + 2 standard deviation scores (SDS) based on the WHO growth charts [28]; gestational age 37–42 weeks; birth weight > 2500 g and < 4000 g; established condition of MUO [29], defined as follows:

- For children ≤ 10 years according to the IDEFICS criteria [waist circumference (WC) ≥ 90th percentile and ≥ 1 of the followings triglycerides (TG) ≥ 90th percentile, HDL cholesterol (HDL) ≤ 10th percentile, systolic blood pressure (SBP) or diastolic blood pressure (DBP) ≥ 90th percentile, HOMA-insulin resistance ≥ 90th percentile or fasting glucose ≥ 90th percentile] [30];
- For children > 10 years according to the IDF criteria [WC ≥ 90th percentile and ≥ 1 of the followings TG ≥ 150 mg/Dl, HDL ≤ 40 mg/dL, SBP ≥ 130 mmHg, DBP ≥ 85 mmHg and fasting blood glucose ≥ 100 mg/dl or known Type 2 Diabetes] [31].

Children were excluded in case of: diagnosis of secondary obesity; treatment with pre/probiotics in the previous 3 months; antibiotic treatment in the previous 3 months; presence of chronic or acute intestinal disease in the previous 3 months.

### Study design

The primary outcome of the project is to evaluate the possible effect of polysaccharide-based complex administration, combined with interventions aimed at improving diet and lifestyle, on gut microbiota composition. This pre–post intervention trial evaluated within-group changes over an 8-month period. Following enrolment (T0), participants underwent a 4-month of a combined dietary intervention and polysaccharide-based complex administration phase (T1). Afterwards, patients underwent a 4-month wash-out phase consisting of dietary and lifestyle intervention alone (T2). Participants attended three study visits (baseline: T0; 4 months: T1; and 8 months: T2). Each participant served as its own control, with comparisons made between T0 vs. T1 vs. T2. At each visit, stool samples were collected, and clinical, anthropometric, dietary, and biochemical assessments were performed.

### Sample size

Since no preliminary data were available on the variability in the gut microbiota composition of the subjects involved in the study, we took as a reference the study by Lammi et al. in which microbial variability was assessed in a pilot study involving 18 children with obesity [32]. In terms of estimating the standard deviation of the outcome, we took as a reference value the maximum standard deviation of the frequency measured for all bacterial genera identified in the study subjects. Without a priori values of Rwithin, a Standard Deviation of the change in the outcome of 0.80 was calculated. Considering the experimental design of the study, we then calculated the sample size using the online tool “Sample size for before-after study (Paired T-test).” We then identified a value of 0.5 for the effect size parameter, and 5% and 20% were identified as alpha and beta error, respectively. On the basis of these data, a sample size of 23 pediatric patients followed at the Vittore Buzzi Children’s Hospital in Milan was assumed to be sufficent. Considering a 20% drop out rate the minimum number of enrolled subjects was 30.

### Intervention

After enrolment at T0, participants were instructed to consume a polysaccharide-based complex (Neo-Policaptil Gel Retard®, European patent no. IT 102020000020467) for 4 months at a dosage of two sachets daily, to be consumed one before lunch and one before dinner (total 5 g/day). This medical device is composed of mixture of soluble and insoluble fibres (cellulose, hemicellulose, pectin, mucilages) from raw materials (glucomannan, cellulose, *Opuntia pulp* stem, chicory root and freeze-dried mallow root, flaxseed and linden flower mucilage) rich in fibres. Compliance to treatment was evaluated by means of a written questionnaire provided to caregivers during visits.

From T0 to T2, participants followed a behaviour lifestyle intervention promoting physical activity and a normocaloric diet based on Italian guidelines for childhood obesity. Dietary recommendations were balanced, age- and sex-adjusted according to the Italian Society of Human Nutrition reference values and aligned with Mediterranean Diet (MD) principles [7, 33].

### Gut microbiota analysis

Stool samples from enrolled patients were collected at T0, T1 and T2 during outpatient visit and immediately stored at -80 °C until analysis. Stool samples analysis was performed at the Pediatric Clinical Research Centre “Fondazione Romeo ed Enrica Invernizzi” Milan (Italy). DNA was extracted from 200 mg of each sample by the QIAamp Fast DNA Stool Mini Kit (Qiagen; Hilden, DE), following the manufacturer’s protocol. The DNA concentration of each sample was assessed fluorometrically. For amplicon production, the V3–V4 hypervariable regions of the prokaryotic 16S ribosomal RNA (rRNA) gene were targeted [34]. PCR was set up in a 50-μl volume with template DNA, 1 × HiFi HotStart Ready Mix (Kapa Biosystems, Wilmington, MA), and 0.5 μM of each primer. The amplification was carried out on a Bio-Rad T100 thermal cycler (Bio-Rad, Hercules, CA) and included: initial denaturation (95◦C for 3 min); 30 cycles at 94◦C for 30 s (s), 55◦C for 30 s, 72◦C for 30 s; and final extension (72◦C for 5 min). Clean-up of amplicons was performed using Agencourt AMPure XP SPRI magnetic beads (ThermoFisher Scientific). Illumina sequencing libraries were finally prepared through the link of indexes (Nextera XT Index Kit, Illumina, San Diego, CA), quantified using aQubit 3 Fluorometer (ThermoFisher Scientific, Waltham,MA), normalized, and pooled. Libraries were subjected to paired-end sequencing (2 × 300 bp format) on an Illumina MiSeq platform at BMR Genomics (Padova, Italy).

### Bioinformatic analysis

The bioinformatic analysis of sequencing data was based on the Mothur pipeline [35]. Briefly, raw FASTQ files were quality-filtered using Trimmomatic [36], and high-quality reads were analyzed following the SOP Mothur procedure. The main ecological indexes of within-sample, alpha diversity (Shannon, Simpson, inverse Simpson, and unbiased Simpson) were computed using Mothur. Microbiota sequencing data were normalized by transforming raw read counts into relative abundances, calculated as the frequency (%) of each taxon over the total number of reads per sample. Diversity in composition among samples (beta diversity) was evaluated at all taxonomic ranks by the relative principal coordinates analysis (PCoA) using the R library Ade4 [37] and Permanova analysis using the R library Vegan [38]. Spearman correlation was performed for a correlation analysis of variables from bacterial frequency at the genus taxonomic level, nutritional data, and SCFA, particularly, butyrate and succinate. A coefficient of ± 0.6–0.8 wass considered as a strong correlation.

### Anthropometry and body composition assessment

At each timepoint, body weight and height were measured with a mechanical column scale and stadiometer. BMI and BMI and related z-scores were calculated using WHO reference growth charts [28]. Qualified nutritionists conducted full anthropometric assessments: body circumferences (mid–upper arm, waist) were measured with a tape, and skinfolds (triceps, subscapular) with a Holtain® caliper. After, z-scores of WC and tricipital skinfold thickness (TSF) were calculated. Waist-to-height ratio (WHtR) was calculated as the ratio between WC and height in centimeters and A Body Shape Index (ABSI) were calculated based on WC adjusted for height and weight to estimate cardiometabolic risk. Body composition was assessed at each timepoint using air-displacement plethysmography (BOD POD®), providing fat mass (FM) and fat-free mass (FFM) in kg and percentage, as well as their respective indexes (FMI and FFMI, respectively).

### Dietary assessment

Food intake at T0, T1, and T2 was assessed using prospective 3-day weighed food records collected with food scales. Caregivers and children/adolescents received instructions to record all foods and beverages consumed over two weekdays and one weekend day. Energy and nutrient intakes were quantified using dedicated software (MètaDieta®). At each visit, adherence to the MD was evaluated with the KIDMED questionnaire, administered by trained staff [39, 40]. The KIDMED index (0–12), derived from 16-question test, classifies adherence into three categories: ≥ 8 optimal adherence to MD, 4–7 improvement needed, and < 3 very low diet quality.

### Clinical examination

Clinical examinations performed at each timepoint included physical examination, Tanner Score evaluation and Bristol Stool Chart identification. Moreover, blood pressure was measured by means of a semiautomatic sphygmomanometer, with two readings per person. In case of medical indication, liver ultrasonography and/or liver magnetic resonance imaging were performed for children and adolescents at risk of metabolic-associated dysfunction steatotic liver disease (MASLD).

### Biochemistry

Hematological and biochemical status was assessed through blood tests performed at T0, T1 and T2, including measurements of fasting glucose, fasting insulin, LDL cholesterol, HDL cholesterol, total cholesterol, triglycerides, aspartate aminotransferase (AST), and alanine aminotransferase (ALT). Insulin sensitivity and insulin-resistance were assessed by calculating the following indexes: HOMA (Homeostasis Model Assessment), HOMA-β (Homeostasis Model Assessment- beta-cell function) and QUICKI (Quantitative Insulin-Sensitivity Check Index) [41, 42]. TyG (Triglyceride–Glucose index), AIP (Atherogenic Index of Plasma) and VAI indexes were calculated [43–45] to evaluate overall cardiometabolic risk.

### Statistical analysis

Continuous variables were checked for normality using the Shapiro–Wilk Test. Prospective changes in anthropometry, body composition, dietary intakes and biochemical variables were tested with one way ANOVA followed by Tukey’s post-hoc test, for normally distributed variables, and with Friedman test and Dunn’s test for pairwise comparison for not-normally distributed variables. For KIDMED score the Wilcoxon matched pairs signed rank test was performed. Taxonomic changes in the gut microbiota were assessed by comparing relative abundances using the Friedman test, followed by Holm–Bonferroni correction for multiple paired comparisons. All results were expressed as the mean ± standard deviation (SD) and p values < 0.05 were considered statistically significant. To assess changes in beta diversity index the microbiota distances were visualized through Principal Coordinates Analysis (PCoA) and the significance threshold (p value) for the PERMANOVA was set < 0.001. Statistical analyses were performed using R_studio (Version 2024.12.0 + 467). Lastly, correlations between gut microbiota, dietary intakes, and SCFAs were assessed using Spearman correlation coefficients.

## Results

### Baseline characteristics

Thirty-six children and adolescents were enrolled, and 31 completed the entire study protocol. Thirty-five patients completed the T1 intervention, with one dropout due to loss of interest. Following this phase, 31 patients completed the T2 intervention and concluded the study, while four participants withdrew primarily due to lack of continued interest or logistical difficulties reaching the hospital.

The cohort included 8 females (26%) and 23 males (74%), with mean age 10.9 ± 1.6 years. At baseline, mean height was 151 ± 12.4 cm, mean weight 68.3 ± 19.1 kg, and mean BMI z-score 3.07 ± 0.5 (Table 1). Over half of participants (54%) met WHO criteria for severe obesity, and 4 presented with MASLD.

**Table 1:** Baseline (T0) characteristics of enrolled children

Baseline variables	
Sex Females Males	*N (%)* 8 (26%) 23 (74%)
	*Mean (SD)*
Age (years)	10.9 (1.6)
Weight (kg)	68.32 (19.11)
Height (cm)	151 (12.38)
BMI (kg/m^2^)	29.39 (4.6)
BMI z-scores	3.07 (0.5)
WC (cm)	93.71 (11.69)
WC z-scores	2 (0.29)
MUAC (cm)	32.62 (4.24)
MUAC z-scores	2.55 (0.45)
Tricep SKF (mm)	30.47 (4.71)
Tricep SKF z-scores	2.25 (0.39)
WHtR	0.62 (0.06)
FM %	42.6 (6.74)
FFM %	57.4 (6.74)
FM (kg)	29.44 (10.68)
FFM (kg)	38.83 (10.8)
FMI	12.64 (3.6)
FFMI	16.65 (2.24)
Systolic blood pressure (mmHg)	114.5 (13.93)
Diastolic blood pressure (mmHg)	74.6 (9.46)
Fasting glucose (mg/dl)	89.27 (6.92)
Fasting Insulin (µU/ml)	25.01 (12.8)
HOMA-IR	5.531 (2.96)
Glycated hemoglobin (mmol/mol)	34.3 (4.46)
LDL cholesterol (mg/dl)	102.5(25.10)
HDL cholesterol (mg/dl)	41.14(7.51)
Total cholesterol (mg/dl)	157.4 (26.5)
Tryglicerides (mg/dl)	118.8 (58.1)
AST (U/l)	26.25 (7.76)
ALT (U/l)	35.71 (25.86)

AST, Aspartate Aminotransferase, ALT, Alanine Aminotransferase; BMI, Body Mass Index; FFM, Fat Free Mass; FFMI, Fat Free Mass Index; FM, Fat Mass; FMI, Fat Mass Index; HDL, High-density lipoproteins cholesterol; HOMA-IR, Homeostatic Model Assessment of Insulin Resistance; kg, kilograms; LDL, Low-density lipoproteins cholesterol, MUAC, Mid-Upper Arm Circumference; SKF, Skinfold; WC, Waist Circumference; WHtR, Waist-to-height Ratio

HOMA-IR was calculated as fasting insulin (µU/ml) × fasting glucose (mg/dL)/405

The baseline prevalence of cardiometabolic alterations, defined according to IDEFICS and IDF criteria, was high: visceral obesity was present in all participants, while hypertriglyceridemia, dyslipidemia, hypertension, and hyperglycemia were observed in 41%, 39%, 23%, and 53% of the cohort, respectively (Table 2).

**Table 2:** Prevalence of metabolically unhealthy obesity (MUO) criteria according to IDEFICS for children 8–10 years and IDF (*) for > 10 years, at T0 (baseline), T1 (4 months, diet + polysaccharide-based complex intervention), and T2 (8 months, diet-only intervention)

	T0 (baseline)	T1 (4 months)	T2 (8 months)
Visceral obesity (WC ≥ 90° percentile)	100%	83%	77%
Hypertriglyceridemia (TG ≥ 90° percentile or TG* ≥ 150 mg/dL)	41%	34%	19%
Dyslipidemia (HDL ≤ 10° percentile or HDL* ≤ 40 mg/dL):	39%	40%	29%
Hypertension (SBP or DBP ≥ 90° percentile or SBP* ≥ 130 mmHg or DBP* ≥ 85 mmHg):	23%	11%	3%
Hyperglycemia (HOMA ≥ 90° percentile or FBG ≥ 90° percentile or FBG* ≥ 100 mg/dl or type 2 diabetes)	53%	37%	32%

FBG, Fasting Blood Glucose; DBP, Diastolic Blood Pressure; HDL, High-density lipoproteins cholesterol; HOMA-IR, Homeostasis Model Assessment of Insulin resistance; LDL, Low-density lipoproteins cholesterol; SBP, Systolic Blood Pressure; TG, Triglycerides; WC, Waist Circumference

### Changes at T1 (4 months) vs. T0 (baseline)

**Fig. 1:**
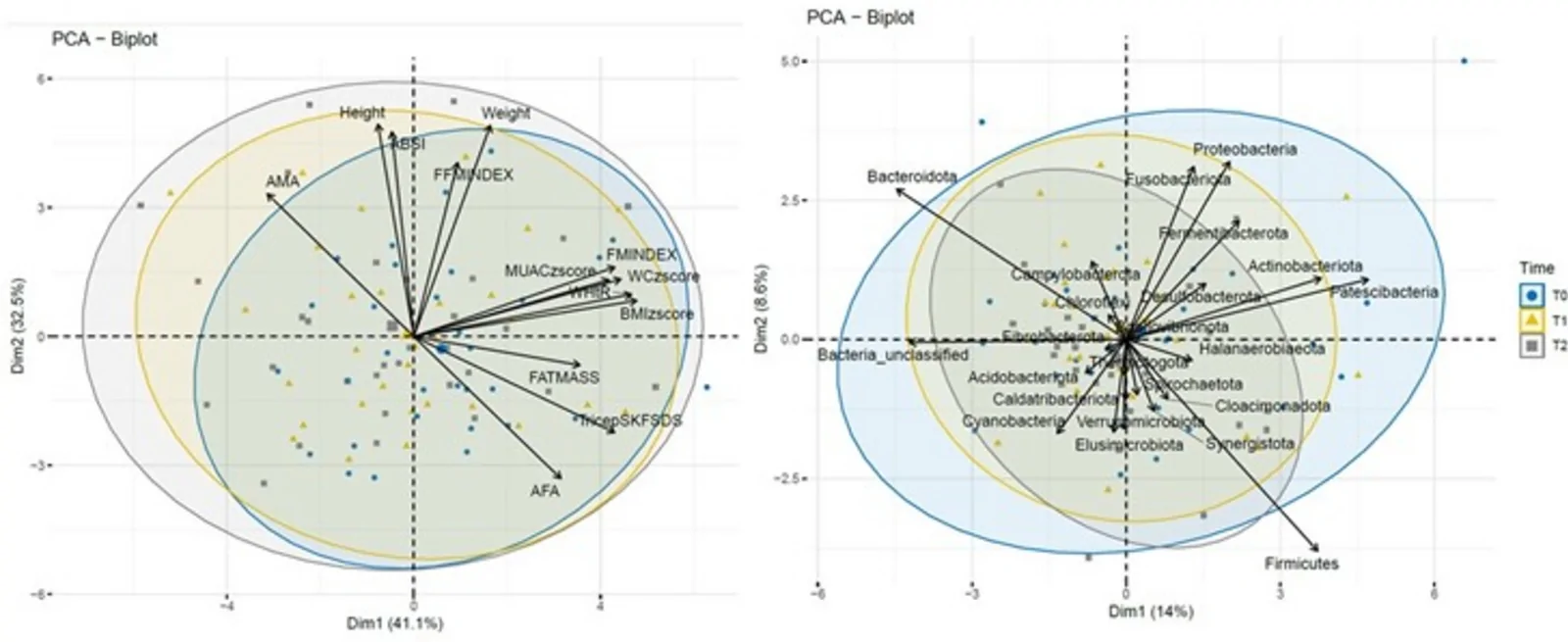
Panel of PCAs: left panel PCA on anthropometry; right panel PCA on gut microbiota at phylum level across different intervention (T0, T1 and T2). Left panel: PCA performed on anthropometric and body composition variables. Right panel: PCA performed on gut microbiota composition at the phylum level. In both panels, each point represents one participant at the corresponding timepoint (T0 = blue, T1 = yellow, T2 = grey). Dim1 (x-axis) and Dim2 (y-axis) represent the first and second principal components, respectively, with the percentage of explained variance indicated in parentheses. Arrows represent the variables contributing to the ordination, with their direction and length reflecting their contribution to the principal components. Ellipses indicate the dispersion of samples within each timepoint group

Figure 1 shows the results of principal component analyses (PCA) for anthropometric variables (left) and gut microbiota composition (right) at T0, T1 and T2. In the anthropometric PCA, the different interventions (T0, T1 and T2) appear partially separated along the first dimension (Dim1, explaining 41.1% of variance), indicating a shift in body composition over time, with reductions in BMI z-score, FM%, and WHtR contributing mostly to the observed changes. Arrows representing anthropometric variables suggest that BMI z-score and FM% decreased after the intervention, whereas height continued to increase with growth.

In the PCA biplot on gut microbiota, the first two dimensions explained 14% and 8.6% of variance, respectively, and show a moderate clustering of samples by timepoint, suggesting that the gut microbial composition shifted at T1. Notably, the arrows indicate that *Firmicutes*, *Bacteroidota* and *Proteobacteria* abundances were major contributors to the variance, implying that the intervention may have modulated key bacterial phyla.

After the T1 intervention, a significant reduction in BMI z-scores (mean percent change -4.50 ± 6.2%; p_*for trend*_ < 0.0001) was observed, together with significant decreases in MUAC z-scores and TSF z-scores (*p*_*for trend*_ < 0.0001 and *p*_*for trend*_ = 0.0002, respectively) (Table 3). Also, WC z-scores (mean percent change -2.83 ± 9.0%) and WHtR (mean percent change -1.54 ± 4.57%) showed a trend of reduction vs. T0, although without reaching statistically significance. A small but significant reduction in FM% (-3.29 ± 8.5%; *p*_*for trend*_ = 0.035) was observed. Paralleling the reduction in FM%, a corresponding increase in FFM% (mean percent change + 2.99 ± 6.1%; *p*_*for trend*_ = 0.035) and FFM in kg (38.8 ± 10.8 at T0 vs. 40.4 ± 11.4 at T1; *p*_*for trend*_ < 0.0001) were found (Table 3). Conversely, there were no significant differences in the T1 values of FM, FMI and FFMI as compared to those at baseline.

**Table 3:** Changes in anthropometric parameters, body composition, clinical and biochemical variables at T0 (baseline), T1 (4 months, diet + polysaccharide-based complex intervention), and T2 (8 months, diet-only intervention)

	T0 (baseline)	T1 (4 months)	T2 (8 months)	p-value for trend (ANOVA)	p-value T0 vs.T1	p-value T1 vs.T2	p-value T0 vs.T2
*Anthropometric parameters, body composition and blood pressure*							
	Mean (SD)	Mean (SD)	Mean (SD)				
Weight (kg)	68.32(19.11)	68.68(18.44)	70.70(19.18)	**0.012**	0.87	**0.0002**	**0.04**
Height (cm)	151(12.38)	152.7(12.14)	154.7(11.97)	**< 0.0001**	**< 0.0001**	**< 0.0001**	**< 0.0001**
BMI (kg/m^2^)	29.39(4.6)	28.97(4.5)	29.05(4.72)	0.27	0.32	0.56	0.89
BMI z-scores	3.07(0.5)	2.94(0.53)	2.85(0.55)	**< 0.0001**	**0.001**	**0.014**	**< 0.0001**
WC (cm)	93.71 (11.69)	93.05 (10.20)	93.95 (11.94)	0.551	0.69	0.51	0.96
WC z-scores	2 (0.29)	1.94 (0.27)	1.9 (0.32)	**0.0053**	0.076	0.46	**0.0093**
MUAC (cm)*	32.62 (4.24)	32.24 (4.26)	31.97 (4.37)	0.089	0.25	0.34	0.15
MUAC z-scores	2.55 (0.45)	2.40 (0.5)	2.28 (0.58)	**< 0.0001**	**0.0027**	0.055	**< 0.0001**
Tricep SKF (mm)	30.47 (4.71)	28.7 (5.49)	28.37 (6.77)	**0.032**	0.069	0.91	0.069
Tricep SKF z-scores	2.25 (0.39)	2.08 (0.46)	2 (0.48)	**0.0002**	**0.023**	0.54	**0.0002**
WHtR	0.62 (0.06)	0.61 (0.06)	0.60 (0.06)	**0.037**	0.13	0.75	**0.05**
ABSI	0.074 (0.009)	0.076 (0.008)	0.077 (0.009)	**0.0013**	**0.0084**	0.32	**0.0028**
FM %	42.6 (6.74)	41.06 (6.37)	41.25 (7.31)	**0.035**	**0.04**	0.92	0.14
FFM %	57.4 (6.74)	58.94 (6.37)	58.75 (7.31)	**0.035**	**0.04**	0.92	0.14
FM (kg)	29.44 (10.68)	28.28 (9.53)	29.25 (9.99)	0.30	0.90	0.61	0.48
FFM (kg)*	38.83 (10.8)	40.37 (11.4)	41.53 (12.15)	**< 0.0001**	**0.033**	0.22	**< 0.0001**
FM index*	12.64 (3.6)	12.03 (3.24)	12.12 (3.43)	0.70	0.9	0.9	0.9
FFM index	16.65 (2.24)	16.98 (2.41)	16.95 (2.74)	0.16	0.16	0.97	0.38
Systolic blood pressure (mmHg)	114.5 (13.93)	115.8 (10.15)	112.7 (12.36)	0.38	0.9	0.67	0.72
Diastolic blood pressure (mmHg)	74.6 (9.46)	71.22 (8.53)	67.5 (8.8)	0.30	0.77	0.53	0.12
*Bristol stool chart Score*							
	Median (IQR)	Median (IQR)	Median (IQR)				
Bristol Stool Chart	3(3;3)	3(3;4)	3(3;3)	0.45	0.9	0.9	0.9
*Biochemical parameters*							
	Mean (SD)	Mean (SD)	Mean (SD)				
Fasting glucose (mg/dl)	89.27 (6.92)	90.39 (6.46)	91.48 (6.44)	0.24	0.35	0.92	0.36
Fasting Insulin (µU/ml)	25.01 (12.8)	24.16 (15.9)	22.85 (13.6)	0.96	0.9	0.9	0.9
HOMA-IR*	5.531 (2.96)	5.464 (3.8)	5.212 (3.34)	0.95	0.9	0.9	0.9
HOMA-beta*	362.7 (188)	329.2 (213.5)	296.2 (160.5)	0.70	0.9	0.9	0.9
QUICKI	0.304 (0.019)	0.309 (0.027)	0.307 (0.022)	0.41	0.4	0.9	0.6
Glycated hemoglobin (mmol/mol)	34.3 (4.46)	33.7 (3.74)	33.9 (3.74)	0.30	0.4	0.9	0.5
LDL cholesterol (mg/dl)	102.5 (25.10)	97.00 (24.71)	93.05 (20.08)	**0.027**	0.83	**0.016**	**0.0008**
HDL cholesterol (mg/dl)	41.14 (7.51)	40.04 (6.58)	40.48 (7.89)	0.33	0.5	0.9	0.85
Total cholesterol (mg/dl)	157.4 (26.5)	149.9 (26.2)	144.7 (23.2)	0.07	0.19	0.39	**0.03**
Tryglicerides (mg/dl)*	118.8 (58.1)	107.5 (48.72)	98.8 (53.2)	0.52	0.9	0.9	0.8
TyG index	4.58 (0.24)	4.54 (0.23)	4.49 (0.23)	0.60	0.9	0.5	0.4
AIP index	0.42 (0.24)	0.4 (0.23)	0.35 (0.26)	0.61	0.9	0.4	0.6
VAI index*	1.78 (1.13)	1.66 (0.92)	1.54 (1.16)	0.60	0.9	0.9	0.9
AST (U/l)*	26.25 (7.76)	24.20 (6.29)	21.85 (6.92)	0.059	0.9	0.14	0.11
ALT(U/l)*	35.71 (25.86)	28.00 (20.45)	24.67 (14.49)	**0.007**	0.84	0.13	**0.006**

*Variables not normally distributed. Statistically significant values (p < 0.05) are shown in bold

ABSI, A-body shape index; AIP index, Atherogenic Index of Plasma; ALT, alanine transaminase; AST, aspartate transaminase; BMI, Body Mass Index; FFM, Fat Free Mass; FM, Fat Mass; HDL, High-density lipoproteins cholesterol; HOMA-IR, Homeostasis Model Assessment of Insulin resistance; LDL, Low-density lipoproteins cholesterol; MUAC, Mid Upper Arm Circumference; QUICKI, Quantitative Insulin-Sensitivity Check Index; Tricep SKF, Tricipital Skinfold Thickness z-score; TyG, Triglyceride–Glucose index; VAI, Visceral Adiposity index; WC, waist circumference; WHtR, Waist-to-height-Ratio

Regarding biochemical parameters, total cholesterol, LDL and triglycerides exhibited a progressive, non-significant decrease over time, while HDL-C remained unchanged (Table 3). Regarding hepatic function, ALT showed a notable downward trend already at T1 (35.7 ± 25.9 U/L at T0 vs. 28.0 ± 20.5 U/L at T1), although without reaching statistical significance.

**Fig. 2:**
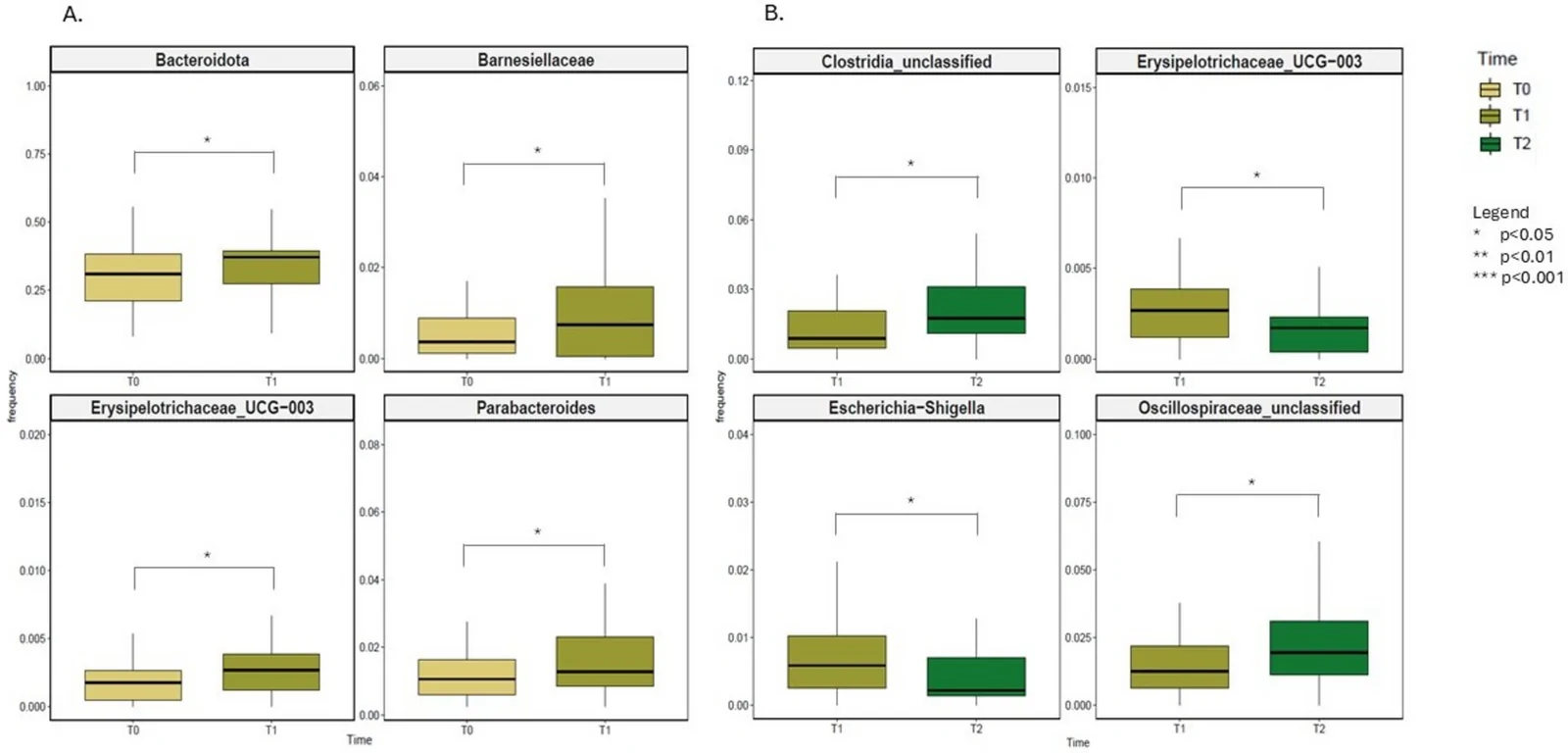
Boxplot panels showing taxonomic changes in gut microbiota relative abundances across intervention phases. **A** Illustrates differences between T0 (baseline) and T1 (4 months; diet plus polysaccharide-based complex intervention), while panel **B** shows differences between T1 and T2 (8 months; diet-only intervention). Taxa are presented at the genus/family level (as indicated in each subplot). Relative abundances are expressed as frequencies. In each boxplot, the central line represents the median, the box indicates the interquartile range (IQR), and whiskers represent the minimum and maximum values

Regarding the gut microbiota, significant increases were detected in *Bacteroidota* at phylum level (p < 0.05). At family level there was an increase in *Barnesiellaceae* abundances (p < 0.05), while at genus level a relevant enrichment in *Erysipelotrichaceae_UCG-003* (p < 0.05), and *Parabacteroides* (p < 0.05) abundances were observed (Fig. 2, Panel A).

### Changes at T2 (8 months) vs. T1 (4 months)

In the PCA on anthropometry, the clustering of samples by intervention (T1 vs. T2) remained close, indicating that most of the variance explained by the PCA was already accounted after the T1 phase. Similarly, in the gut microbiota PCA, the ordination plots suggested stability in microbial community structure, without further separation, indicating that the most relevant compositional shift had occurred during the T1 phase.

A significant decrease in BMI z-scores was observed at T2 vs. T1 (mean percent change -3.09 ± 6.1%; *p*_*for trend*_ < 0.0001), confirming the maintenance of obesity reduction over time. Waist circumference z-scores, and WHtR, did not change significantly (Table 3). Regarding body composition, FM% remained unchanged (mean percent change + 0.2 ± 7.3%; no significant difference), while FFM, expressed as absolute value (in kg), continued to increase (40.37 ± 11.4 at T1 vs. 41.53 ± 12.15 at T2), indicating a physiological gain in lean tissue associated with growth, although without statistical significance.

Regarding biochemical parameters, LDL cholesterol was significantly reduced (97.00 ± 24.71 at T1 vs. 93.05 ± 20.08 at T2, p_*for trend*_ = 0.027), and the trend of reduction in ALT levels continued, although without statistical significance.

At gut microbiota level, no major changes were observed at phylum level. At the genus level, significant changes were observed between T1 and T2. Specifically, the relative abundance of *Oscillospiraceae_unclassified* and *Clostridia_unclassified*, increased significantly (p < 0.05), whereas *Erysipelotrichaceae_UCG − 003* and *Escherichia_Shigella* decreased signifcantly (p < 0.05) (Fig. 2, Panel B).

### Overall trend (T2 vs. T0)

Anthropometrically, BMI z-scores decreased significantly from baseline to end of the intervention (mean percent change -7.51 ± 7.6%, p < 0.0001). The mean absolute reduction in BMI z-scores after 8 months was − 0.22 ± 0.23; notably, five participants were classified as non-responders, as they did not exhibit any reduction in BMI z-scores at the end of the intervention. Significant reduction were observed for WC z-scores (mean percent change -4.89 ± 10.15%, *p*_*for trend*_ = 0.0053) and WHtR (mean percent change -2.41 ± 5.53%, *p*_*for trend*_ = 0.037), indicating progressive reduction in visceral adiposity (Table 3). At the end of the study, no major changes in FM% and kg or FMI were observed. Regarding biochemical parameters, LDL cholesterol decreased progressively from 102.5 ± 25.10 mg/dL at T0 to 93.05 ± 20.08 mg/dL at T2 (p_*for trend*_ = 0.027), and ALT declined from 35.71 ± 25.86 U/L at T0 to 24.67 ± 14.49 U/L at T2 (p_*for trend*_ = 0.007), while other parameters including fasting glycemia, fasting insulin, HOMA-IR, HDL, triglycerides, and AST showed non-significant changes. However, when analyzed according to categories of metabolic alterations, the prevalence of hypertriglyceridemia, hypertension, and hyperglycemia decreased notably, all over the study period (Table 2).

Gut microbiota analysis revealed a significant decrease in the phylum *Proteobacteria* abundances over time (p < 0.05). At finer taxonomic levels a reduction in *Enterobacteriaceae* and *Clostridia_UCG − 014* abundances were observed, as well as a reduction in *Escherichia − Shigella* over time (p < 0.05), indicating a decrease in potentially pathogenic taxa (Supplementary Table 1).

No major changes were observed for alpha diversity indexes (Chao, Shannon, Inverted Simpson, Shannon evenness) over the entire study period (Supplementary Fig. 1). Also, SCFA abundances, namely butyrate and succinate, did not significantly change over time (Supplementary Fig. 2).

### Nutritional intakes

Intakes of energy, macronutrients and micronutrients over the study period are presented in Table 4.

**Table 4:** Energy and nutrient intakes at T0 (baseline), T1 (4 months, diet + polysaccharide-based complex intervention), and T2 (8 months, diet-only intervention)

	T0 (baseline)	T1 (4 months)	T2 (8 months)	p-value for trend (ANOVA)	p-value T0 vs.T1	p-value T1 vs.T2	p-value T0 vs.T2
Energy (Kcal)	1871(303)	1669(390)	1797(322)	**0.003**	**0.002**	0.4	0.2
Carbohydrates (% En)*	48(5.8)	46(5.2)	50(6.8)	0.057	0.5	0.08	0.3
Carbohydrates (g)	240(54)	209(63)	240(55)	**0.008**	**0.01**	**0.04**	0.9
Sugars (%En)	13.2(3.7)	13.4(3.1)	14.4(6.4)	0.968	0.9	0.9	0.9
Sugars (g)	65(20.7)	58(25)	66(36)	**0.027**	**0.02**	0.4	0.8
Dietary fibres (g)	13.21(6.1)	14.8(5.8)	17(5.6)	**0.018**	0.9	0.2	**0.02**
Soluble fibers (g)	2.1(1.2)	2.2(1.1)	2.4(1.2)	0.856	0.9	0.9	0.9
Insoluble fibers (g)	5.5(4.3)	6.0(3.1)	6.7(4.1)	0.405	0.9	0.9	0.6
Lipids (% En)	35.9(5.5)	35.9(4.7)	33.9(8.6)	0.405	0.9	0.9	0.6
Lipids (g)	74.4(15.2	65.8(14.3)	67.5(18.8)	0.089	0.2	0.9	0.2
Saturated fatty acids (% En)*	10.1(2.7)	9.8(2.3)	9.5(3.2)	0.728	0.9	0.9	0.7
Saturated fatty acids (g)	20.8(6.2)	18.3(6.3)	19.3(7.7)	0.303	0.4	0.9	0.6
Monounsaturated fatty acids (% En)*	13.7(2.8)	15.1(3.7)	13.8(3.4)	0.112	0.2	0.2	0.8
Monounsaturated fatty acids (g)	27.9(5.2)	27.4(6.6)	26.9(5.8)	0.657	0.9	0.9	0.9
Polyunsaturated fatty acids (%En)	4.2(1.7)	4.2(1.3)	3.8(1.2)	**< 0.0001**	0.9	**< 0.0001**	**< 0.0001**
Polyunsaturated fatty acids (g)	8.7(3.9)	7.6(3)	7.4(2.4)	0.276	0.9	0.9	0.3
Proteins (%En)	15.3(3)	16.5(3.6)	15.7(2.1)	0.159	0.2	0.7	0.9
Proteins (g) *	71.1(15.5)	68.9(21.6)	69.9(13.8)	0.790	0.8	0.9	0.9
Animal proteins (g) *	41.1(15.4)	42.9(18.7)	41.4(10.5)	0.787	0.8	0.8	0.9
Plant proteins (g) *	19.4(7.3)	19.04(6)	21.2(7.1)	0.381	0.9	0.3	0.6
Omega 3 (%En)	0.44(0.2)	0.48(0.2)	0.53(0.2)	0.094	0.7	0.9	0.09
Omega 6 (%En)	3.4(1.6)	3.3(0.9)	2.8(1.0)	0.541	0.9	0.9	0.9
Vitamin B12 (μg)	3.4(4.0)	3.2(1.7)	5.4(10)	0.418	0.9	0.7	0.7
Folic acid (μg)	197(91.3)	220(109.5)	257(109.9)	0.053	0.9	0.5	**0.04**
Vitamin A (μg)	584(452)	687(414)	898(1537)	0.748	0.9	0.9	0.9
Vitamin E (mg)	9.9(3.2)	10.2(3.6)	10(3.7)	0.657	0.9	0.9	0.9
Calcium (mg)	543(233)	503(240)	531(244.5)	0.596	0.9	0.9	0.9
Iron (mg)*	7.7(3)	8.4(3.4)	9.4(3.86)	0.073	0.5	0.4	0.09
Zinc (mg)*	7.5(2.4)	7.8(2.7)	8.2(1.9)	0.256	0.8	0.6	0.2
Magnesium (mg)	142(52.4)	167(69.5)	159(58.5)	0.541	0.9	0.9	0.9

Variables not normally distributed. Statistically significant values (p < 0.05) are shown in bold

%En. percentage of the energy intake

There was a significant decrease in the estimated energy intake at T1 (from 1871 to 1669 kcal, p_*for trend*_ = 0.003), whilst at the end of the study period there was a slight increase in energy intake, although without statistically significant difference compared to T0 and T1. Carbohydrate intake, expressed in grams but not as a percentage of total energy, significantly decreased at T1 compared with both T0 and T2. A similar trend was observed for sugar intake, which decreased in grams but not in percentage. By the end of the study, sugar intake returned to absolute values comparable to baseline.

Regarding dietary fibres intake, there was a significant increase in dietary intakes (g/day) only at the end of the study compared to baseline (p_*for trend*_ = 0.018). At T1 intake of fibres slightly increased from baseline, without statistical significance. No significant differences were observed in the amounts of soluble and insoluble fibres, although the mean intake increased. There are no further significant differences in nutritional intakes, other than a reduction in the relative intake of polyunsaturated fatty acids at T2 vs. T1.

Regarding adherence to the MD, we observed a significant increase in KIDMED score at T1 (from a median score of 2 to 5, p_*for trend*_ < 0.0001), passing from a very low diet quality to a medium adherence to MD. At T2 KIMED score remained constant with no decreasing trend (Table 5).

**Table 5:** KIDMED score at T0 (baseline), T1 (4 months, diet + polysaccharide-based complex intervention), and T2 (8 months, diet-only intervention). Statistically significant values (p < 0.05) are shown in bold

	T0 (baseline) Median(IQR)	T1 (4 months) Median(IQR)	T2 (8 months) Median(IQR)	p-value for trend (ANOVA)	p-value T0 vs.T1	p-value T1 vs.T2	p-value T0 vs.T2
KIDMED score	2(1;4)	5(2;6)	5(3;6)	**< 0.0001**	**0.0085**	0.84	**0.001**

**Fig. 3:**
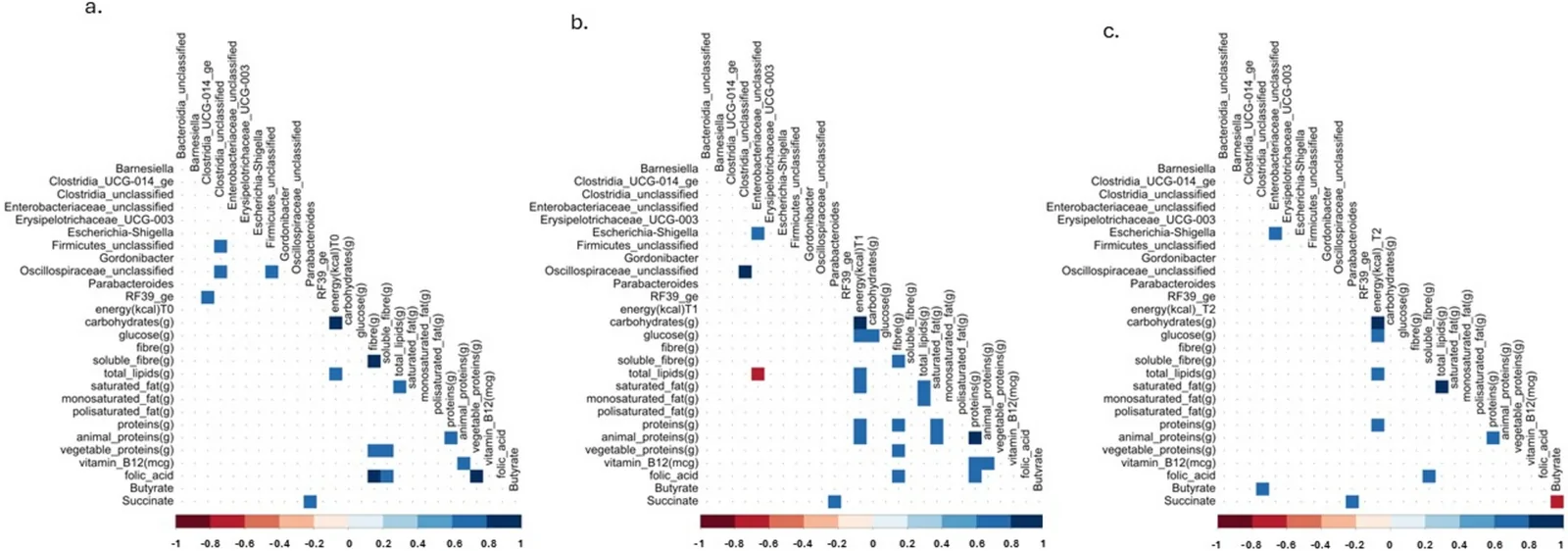
Correlation matrices between dietary intakes, gut microbiota composition, and short-chain fatty acids (SCFAs) at different timepoints. **A**: T0 (baseline); **B**: T1 (4 months); **C**: T2 (8 months). Spearman’s correlation analysis was performed to assess associations between bacterial relative abundances at the genus level, nutritional intake variables, and estimated SCFA concentrations. Correlation coefficients are represented according to colour intensity and direction, indicating the strength and polarity of associations

Correlation matrices were generated to explore the associations between dietary intakes and gut microbiota composition across the three timepoints (Fig. 3). We primarily observed a strong negative correlation between total lipids and *Enterobacteriaceae_unclassified* at T1.

## Discussion

In our intervention study we aim to assess the effect of polysaccharide-based complex administration, combined with a dietary intervention, on the gut microbiota composition, obesity status and cardio-metabolic parameters of a cohort of paediatric patients with obesity and MUO.

From a multivariate perspective, the PCA plots support a noticeable shrinkage of variance in gut microbiota ordinations, compared with baseline, after diet with polysaccharide-based complex intervention (i.e. T1), suggesting a reduction in individual dispersion and a transition toward a less chaotic metabolic–microbial environment. This pattern implies a more homogeneous response among participants, likely reflecting stabilization of microbial profiles following the combined intervention. The diet with polysaccharide-based complex intervention phase thus appears to act as the primary trigger for these coordinated shifts, while the subsequent solo-diet phase keeps stable this configuration with less divergence. At the phylum level, these trends were characterized by an expansion of *Bacteroidota* at T1 and a progressive reduction of *Proteobacteria* at T2 (the end of the study), consistent with an overall shift toward a healthier gut microbial composition.

Specifically, at T1 participants exhibited a significant decrease in BMI z-scores and FM%, paralleled by specific shifts in gut microbiota taxa. Notably, increases in *Parabacteroides* and *Barnesiellaceae* abundances were observed. *Parabacteroides* has been inversely associated with obesity and metabolic impairment, being considered a genus in close relationship with host health [46, 47]. Similarly, we observed an enrichment in *Barnesiellaceae* after treatment. Interestingly, Del Chierico et al. previously defined *Barnesiellaceae* and *Parabacteroides* as a distinct microbial marker of adolescence gut microbiota distinguishing normal weight from adolescents with obesity and MASLD [48, 49]. *Barnesiella* (belonging to *Barnesiellaceae* family) enrichment was also outlined in an adult cohort of healthy individuals compared to patients with obesity [50].

The concurrent decrease in adiposity and enrichment of taxa typically associated with metabolic health suggests that diet + polysaccharide-based complex intervention may have promoted a favourable metabolic-microbial axis, possibly mediated by polysaccharide-based complex administration.

In contrast, between T1 and T2 (diet-solo phase), microbial changes appeared less pronounced in terms of community structure but revealed an increase in *Oscillospiraceae* family. *Oscillospiraceae* are well-known butyrate producers and have been negatively associated with obesity and inflammatory bowel disease [51–53]. The expansion of this butyrate-producing lineages in the absence of polysaccharide-based complex administration indicates that the dietary changes were the predominant driver for this shift. Butyrate is the SCFA that plays the most important role in physiological functions and protection against several cardiometabolic alterations related to obesity [54, 55]. Specifically, butyrate is the main source of energy for colonocytes, and it has well-documented roles in maintaining gut barrier integrity, modulating inflammation and cytokine expression [56], suggesting that these changes may further reinforce the metabolic improvements observed throughout the intervention.

Among the changes observed at the end of the study period, it is noteworthy the reduction in *Enterobacteriaceae* family, compared to baseline . Cross-sectional studies on adults have shown that individuals with obesity, overweight, and metabolic syndrome have a significantly higher abundance of *Enterobacteriaceae* compared to normal-weight controls [57, 58]. Similarly, in preschool-aged children (4–5 years), the abundance of *Enterobacteriaceae* is significantly higher in subjects with obesity and overweight [59]. The alteration of oxygen levels in the lumen, due to microbial changes associated with obesity (such as a reduction in butyrate-producing bacteria) would, in turn, favour an increase in facultative anaerobic bacteria like *Enterobacteriaceae* [58]. Although normal-weight controls were not included, *Enterobacteriaceae* abundances were high at baseline and declined during follow-up, possibly reflecting the underlying improvement in obesity status of the enrolled children. Our study aligns with a study on adult patients with obesity and MASLD, where the abundance of *Enterobacteriaceae* significantly decreased following a weight-reduction dietary intervention [60]. The reduction in the relative abundance of *Enterobacteriaceae* is likely the result of the improvement in dietary quality that goes along with the significant reduction in pathogenic taxa reduction such as *Escherichia − Shigella* over time.

Interestingly, alpha-diversity metrics remained stable across all the study, indicating that the intervention influenced the taxonomic composition rather than the overall richness or evenness of the microbial community. Indeed, the observed associations raise the possibility that changes in body composition (specifically reductions in BMI z-scores and FM%) could act as primary drivers of microbiota modulation in children and adolescents with obesity, consistent with recent evidence suggesting bidirectional interactions between adiposity and microbial metabolism [61].

Anthropometric parameters showed that during all the study period there was a trend towards normalization of weight gain and a reduction in BMI z-scores. Interestingly we observed a significant reduction in FM% at T1, thus pointing to the efficacy of diet combined with polysaccharide-based complex in reducing fat mass compared to diet alone. These results might be driven by the already reported fibre satiety role [25, 26]. Overall, the improvement in adiposity may have positively influenced the improvement trends in glucose and lipid profiles, although without significance. There was only a significant reduction in LDL cholesterol and ALT at the end of the study, which could be the result of the overall downward trend observed throughout the study period supporting the beneficial metabolic trajectory associated with the intervention. We acknowledge that the sample size was not estimated a priori for biochemical endpoints; hence the study had limited power to detect meaningful differences in biochemical parameters. By administering polysaccharide-based complex with low-glycaemic index diet in children with obesity, Stagi et al. showed a greater BMI and glycated haemoglobin reduction, compared to diet-alone group [27]. They also observed changes in the glucose metabolism, although without superiority compared to control group. It should be noted that different effects on metabolic parameters could be ascribed to different lifestyle intervention, i.e. interventions focused on exercise and/or cognitive-behavioural therapy, in addition to diet.

Nutritional-behavioural interventions are the cornerstone of childhood obesity management [6–8]. In addition, emerging evidence suggests that childhood obesity and its metabolic consequences may differently affect males and females, including endocrine and developmental outcomes, highlighting the importance of incorporating sex- and gender-based approaches into paediatric nutrition [62–64]. Furthermore, epidemiological data from adult populations demonstrate a synergistic effect of increased BMI and impaired glucose metabolism on cardiovascular events and all-cause mortality, with partially sex-specific risk patterns, reinforcing the importance of early and personalized prevention strategies [65].There is growing interest in complementing dietary and lifestyle interventions for the treatment of childhood obesity. More recently, glucagon-like peptide-1 (GLP-1) receptor agonists, notably semaglutide, have been introduced for paediatric obesity management [66, 67]. GLP-1 receptor agonists have demonstrated significant efficacy in adult obesity, not only in promoting weight loss but also in improving glycaemic control and reducing cardiometabolic risk [68, 69]. In addition, these agents have shown cardio-renal protective and anti-inflammatory effects [70].

Importantly, evidence suggests that weight loss per se, regardless of the intervention strategy, is associated with improvements in inflammatory status. In adults with obesity, weight loss greater than 5% has been associated with improvements in inflammatory markers, particularly interleukin-6 [71], while in paediatric populations, moderate weight reduction has also been linked to amelioration of obesity-related low-grade inflammation [72]. However, the inflammatory response to weight loss is complex, and a broader panel of inflammatory markers should be considered to better characterize these effects [73], without overlooking the role of diet per se [74].

Maintaining long-term weight loss after pharmacological treatment discontinuation is challenging due to metabolic adaptation and the challenges in adhering to lifestyle interventions, with weight often being regained following initial weight loss [75]. These challenges prompt investigation into whether administration of polysaccharide-based complexes (either following pharmacological treatment or given concomitantly at targeted doses) may enhance or prolong favourable metabolic and anthropometric outcomes.

Some limitations must be acknowledged in our study. Firstly, dietary changes undertaken by patients between T0 and T1 remain a confounding factor. Nonetheless, enrolling patients with obesity without any treatment would not be ethical. Thus, an experimental design comprising an intervention and a control diet period allows patients to act as their own controls at the end of the intervention, increasing the reliability of the results in the absence of a true control group. Additionally, the sample size and study duration limit the generalizability of the findings. The absence of an intention-to-treat analysis, due to missing follow-up data in participants who withdrew, represents a study limitation and may have introduced attrition bias. However, to our knowledge this is the first study to assess the impact of a polysaccharide-based complex on the gut microbiota of children and adolescents with metabolically unhealthy obesity. The body composition assessment undoubtedly represents a strength of the present study, since air plethysmography (BOD POD) directly measures body density without any invasiveness, being able to more accurately estimate fat mass and consequently body composition, compared to other less precise estimation methodologies affected by confounding factors.

In conclusion, this study suggests that a combined dietary and polysaccharide-based complex intervention can promote reduction in body adiposity and favourable gut microbiota changes in children and adolescents with obesity. The enrichment observed at different level indicates that both interventions may synergistically contribute to the metabolic improvement via distinct microbial pathways. Future studies incorporating longer follow-up and functional metagenomic analyses of gut microbiota are warranted to elucidate causal mechanisms and optimize personalized dietary–microbiota interventions in childhood obesity.

## Supplementary Information

Below is the link to the electronic supplementary material.

Supplementary file1 (PDF 6 KB). Supplementary Figure 1: Boxplot of alpha diversity indexes (Chao, Simpson, Shannon, Shannon eveness) at T0 (baseline), T1 (diet + polysaccharide-based complex intervention), and T2 (diet-only intervention).

Supplementary file2 (PDF 51 KB). Supplementary Figure 2: Violin plot of estimated SCFA abundances (i.e. succinate and butyrate) at T0 (baseline), T1 (diet + polysaccharide-based complex intervention), and T2 (diet-only intervention).

Supplementary file3 (PDF 139 KB). Supplementary Figure 3: Rarefaction curves showing the number of OTUs as a function of the number of sequences.

Supplementary file4 (DOCX 1649 KB). Supplementary Table 1: Observed taxonomic changes in gut microbiota abundances at different timepoints: T0 (baseline), vs. T1 (4 months, diet + polysaccharide-based complex intervention), v. T2 (8 months, diet-only intervention). Analysis were performed according to Friedman test, followed by Holm–Bonferroni correction for multiple paired comparisons

## Declarations

### Data availability statement

The clinical and patient data supporting the findings of this study are not publicly available due to their sensitive nature but are available from the corresponding author upon reasonable request.
