# Supplementary material for: Synergic effect of diet and polysaccharide-based complex administration in the treatment of children and adolescents with metabolically unhealthy obesity: the Polymets Study
Source: J Endocrinol Invest. 2026 Mar 14;49(9):2461–75. doi: 10.1007/s40618-026-02860-0 (PMC13498496; doi:10.1007/s40618-026-02860-0)
Supplement: Supplementary file 1 [file 40618_2026_2860_MOESM1_ESM.pdf]

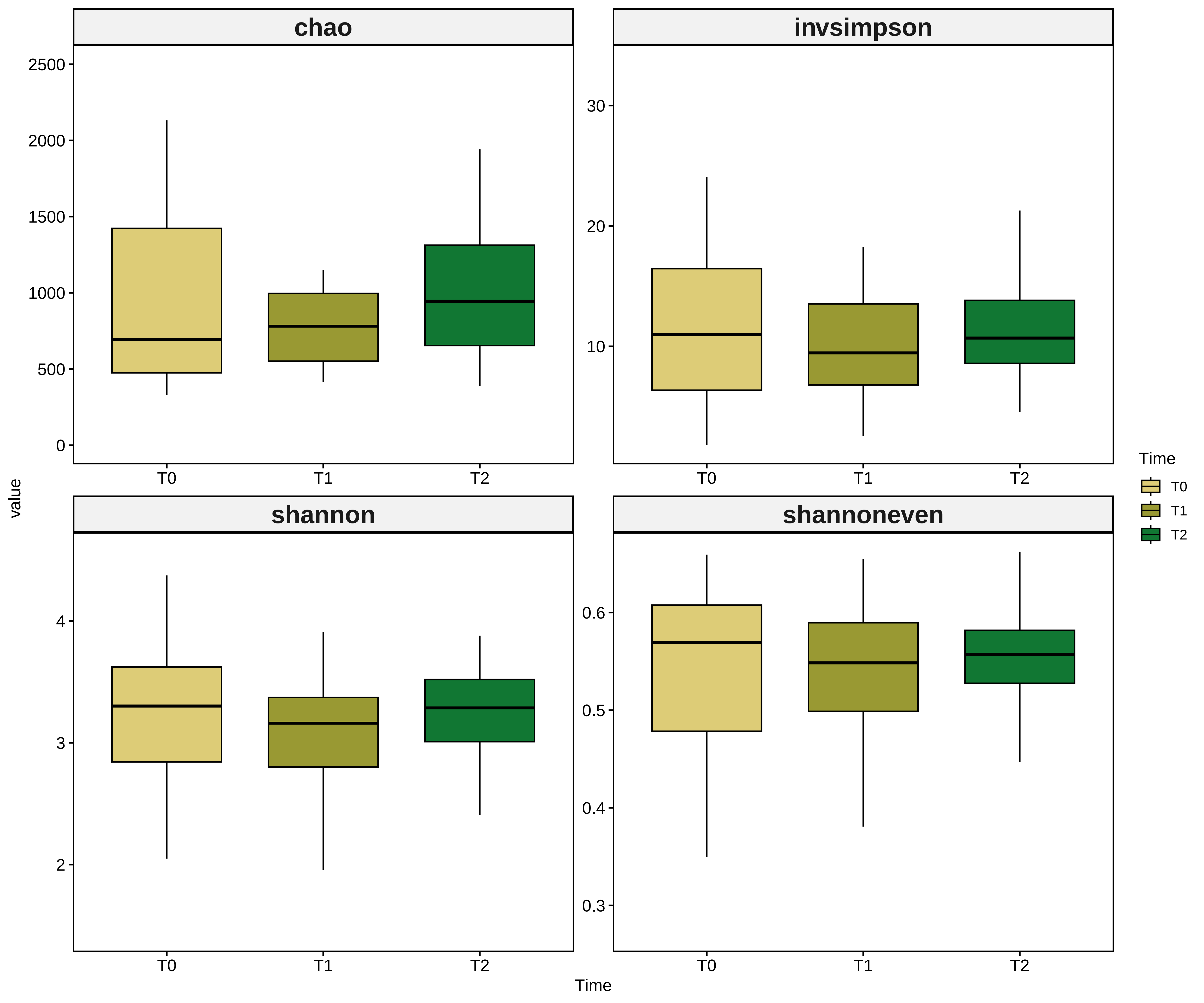
chao
invsimpson
shannon
shannoneven
value
Time
T0
T1
T2
0
500
1000
1500
2000
2500
10
20
30
2
3
4
0.3
0.4
0.5
0.6
