# Supplementary material for: Synergic effect of diet and polysaccharide-based complex administration in the treatment of children and adolescents with metabolically unhealthy obesity: the Polymets Study
Source: J Endocrinol Invest. 2026 Mar 14;49(9):2461–75. doi: 10.1007/s40618-026-02860-0 (PMC13498496; doi:10.1007/s40618-026-02860-0)
Supplement: Supplementary file 2 [file 40618_2026_2860_MOESM2_ESM.pdf]

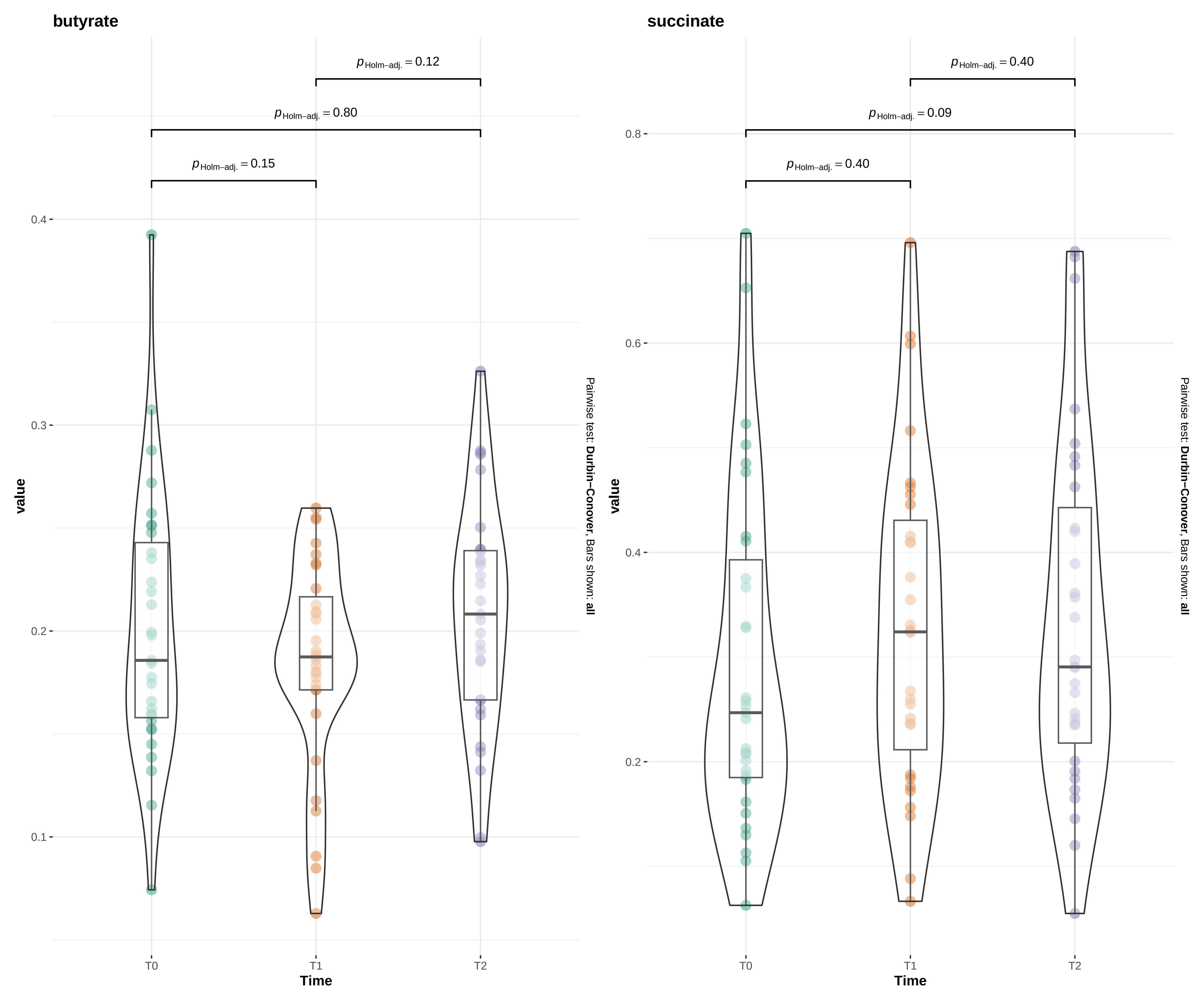

butyrate
$p_{Holm-adj.} = 0.12$
$p_{Holm-adj.} = 0.80$
$p_{Holm-adj.} = 0.15$
value
0.4
0.3
0.2
0.1
T0
T1
T2
Time
Pairwise test: **Durbin-Conover**, Bars shown: **all**
succinate
$p_{Holm-adj.} = 0.40$
$p_{Holm-adj.} = 0.09$
$p_{Holm-adj.} = 0.40$
value
0.8
0.6
0.4
0.2
T0
T1
T2
Time
Pairwise test: **Durbin-Conover**, Bars shown: **all**
