# Supplementary material for: Synergic effect of diet and polysaccharide-based complex administration in the treatment of children and adolescents with metabolically unhealthy obesity: the Polymets Study
Source: J Endocrinol Invest. 2026 Mar 14;49(9):2461–75. doi: 10.1007/s40618-026-02860-0 (PMC13498496; doi:10.1007/s40618-026-02860-0)
Supplement: Supplementary file 3 [file 40618_2026_2860_MOESM3_ESM.pdf]

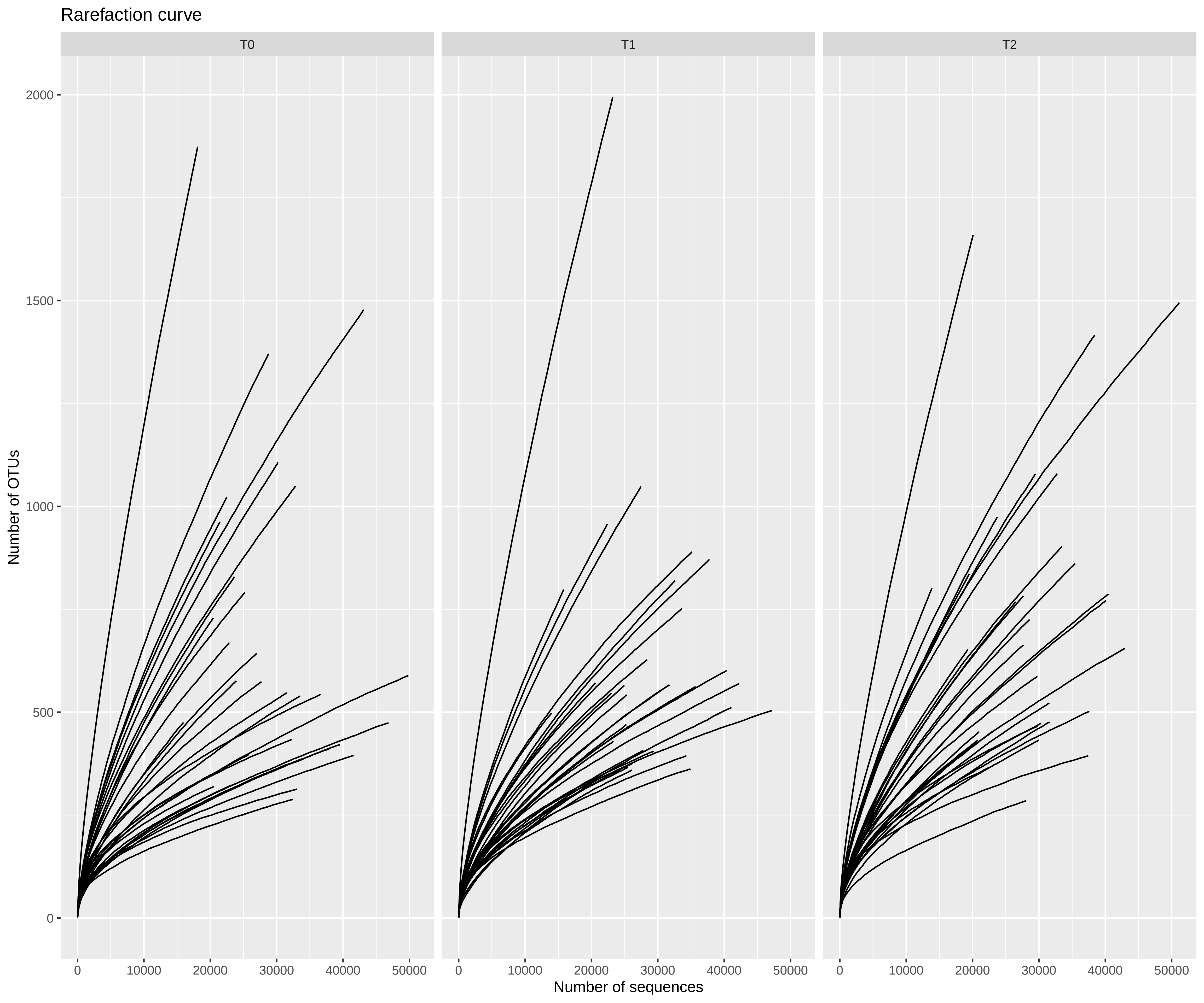

Rarefaction curve
T0
T1
T2
Number of OTUs
0
500
1000
1500
2000
0
10000
20000
30000
40000
50000
Number of sequences
