# Supplementary material for: Synergic effect of diet and polysaccharide-based complex administration in the treatment of children and adolescents with metabolically unhealthy obesity: the Polymets Study
Source: J Endocrinol Invest. 2026 Mar 14;49(9):2461–75. doi: 10.1007/s40618-026-02860-0 (PMC13498496; doi:10.1007/s40618-026-02860-0)
Supplement: Supplementary file 4 [file 40618_2026_2860_MOESM4_ESM.docx]

Supplementary Table 1. Observed taxonomic changes in gut microbiota abundances at different timepoints: T0 (baseline), vs. T1 (4 months, diet + polysaccharide-based complex intervention), v. T2 (8 months, diet-only intervention). Analysis were performed according to Friedman test, followed by Holm–Bonferroni correction for multiple paired comparisons.

| **Bacteria** | **Tax. level** | **T0**  **Median(IQR)** | **T1**  **Median(IQR)** | **T2**  **Median(IQR)** | **P-value**  **(T0-T1)** | **P-value**  **(T1-T2)** | **P-value**  **(T0-T2)** |
| --- | --- | --- | --- | --- | --- | --- | --- |
| Bacteroidota | phylum | 0.309  (0.173)^a^ | 0.372  (0.121)^b^ | 0.319  (0.115)^a,b^ | 0.017 |  |  |
| Proteobacteria | phylum | 0.046  (0.07)^a^ | 0.051  (0.053)^a,b^ | 0.038  (0.032)^b^ |  |  | 0.017 |
| Firmicutes_unclassified | class | 0.003  (0.009)^a^ | 0.005  (0.006)^a^ | 0.009  (0.003)^b^ |  | 0.002 | 0.009 |
| Bacteroidales | order | 0.305  (0.164)^a^ | 0.363  (0.123)^b^ | 0.317  (0.117)^a,b^ | 1.2e^-2^  (0.037) |  |  |
| Bacteroidia_unclassified | order | 0.003  (0.007)^a,b^ | 0.003  (0.004)^a^ | 0.008  (0.007)^b^ |  | 6.89e^-4^  (0.002) |  |
| Clostridia_UCG-014 | order | 0.001  (0.009)^a^ | 0(0.007)^a,b^ | 0(0.003)^b^ |  |  | 6e^-3^  (0.018) |
| Clostridia_unclassified | order | 0.014  (0.018)^a,b^ | 0.008  (0.016)^a^ | 0.017  (0.02)^b^ |  | 9e^-3^  (0.026) |  |
| Enterobacterales | order | 0.025  (0.042)^a^ | 0.022  (0.033)^a,b^ | 0.016  (0.019)^b^ |  |  | 3e^-3^  (0.009) |
| Firmicutes_unclassified | order | 0.003  (0.009)^a^ | 0.005  (0.006)^a^ | 0.009  (0.013)^b^ |  | 2e^-3^  (0.007) | 9e^-3^  (0.018) |
| RF39 | order | 0(0.0003)^a,b^ | 0(0)^a^ | 0(0.001)^b^ |  | 1.4e^-2^  (0.043) |  |
| Bacteroidia_unclassified | family | 0.003  (0.007)^a,b^ | 0.003  (0.004)^a^ | 0.008  (0.007)^b^ |  | 6.89e^-4^  (0.002) |  |
| Barnesiellaceae | family | 0.003  (0.007)^a^ | 0.007  (0.015)^b^ | 0.004  (0.01)^a,b^ | 6e^-3^  (0.019) |  |  |
| Clostridia_UCG-014_fa | family | 0.001  (0.009)^a^ | 0(0.007)^a,b^ | 0(0.003)^b^ |  |  | 6e^-3^  (0.018) |
| Clostridia_unclassified | family | 0.014  (0.018)^a^ | 0.008  (0.016)^b^ | 0.017  (0.02)^a,b^ |  | 9e^-3^  (0.026) |  |
| Enterobacteriaceae | family | 0.01  (0.03)^a^ | 0.009  (0.017)^a,b^ | 0.005  (0.012)^b^ |  |  | 9.04e^-4^  (0.003) |
| Firmicutes_unclassified | family | 0.003  (0.009)^a^ | 0.005  (0.006)^a^ | 0.009  (0.013)^b^ |  | 2e^-3^  (0.007) | 9e^-3^  (0.018) |
| Oxalobacteraceae | family | 0(0)^a^ | 0(0.0005)^b^ | 0(0)^a,b^ | 1.4e^-2^  (0.043) |  |  |
| RF39_fa | family | 0(0.0003)^a,b^ | 0(0)^a^ | 0(0.0017)^b^ |  | 1.4e^-2^  (0.043) |  |
| Bacteroidia_unclassified | genus | 0.003  (0.007)^a,b^ | 0.003  (0.004)^a^ | 0.008  (0.007)^b^ |  | 6.89e^-4^  (0.002) |  |
| Barnesiella | genus | 0.002  (0.006)^a^ | 0.005  (0.011)^b^ | 0.004  (0.008)^a, b^ | 9e^-3^  (0.028) |  |  |
| Clostridia_UCG-014_ge | genus | 0.001  (0.009)^a^ | 0(0.007)^a,b^ | 0(0.003)^b^ |  |  | 6e^-3^  (0.018) |
| Clostridia_unclassified | genus | 0.014  (0.018)^a,b^ | 0.008  (0.016)^a^ | 0.017  (0.02)^b^ |  | 9e^-3^  (0.026) |  |
| Enterobacteriaceae_unclassified | genus | 0.004  (0.014)^a^ | 0.002  (0.008)^a,b^ | 0.002  (0.004)^a^ |  |  | 1.1e^-2^  (0.032) |
| Erysipelotrichaceae_UCG-003 | genus | 0.001  (0.002)^a^ | 0.002  (0.002)^b^ | 0.001  (0.001)^a^ | 2.1e^-2^ (0.041) | 1.1e^-2^  (0.033) |  |
| Escherichia_Shigella | genus | 0.005  (0.011)^a^ | 0.005  (0.007)^a^ | 0.002  (0.005)^b^ |  | 7e^-3^  (0.021) | 8e^-3^  (0.021) |
| Firmicutes_unclassified | genus | 0.003  (0.009)^a^ | 0.005  (0.006)^a^ | 0.009  (0.013)^b^ |  | 2e^-3^  (0.007) | 9e^-3^  (0.018) |
| Gordonibacter | genus | 0(0.001)^a^ | 0(0)^b^ | 0(0)^a,b^ | 1.7e^-2^  (0.007) |  |  |
| Oscillospiraceae_unclassified | genus | 0.014  (0.015)^a,b^ | 0.012  (0.015)^a^ | 0.019  (0.019)^b^ |  | 5e^-3^  (0.015) |  |
| Parabacteroides | genus | 0.01  (0.01)^a^ | 0.01  (0.014)^b^ | 0.011  (0.011)^a,b^ | 1e^-2^  (0.031) |  |  |
| RF39_ge | genus | 0(0.0003)^a,b^ | 0(0)^a^ | 0(0.0017)^b^ |  | 1.4e^-2^  (0.043) |  |
